# Supplementary material for: Comparison of osteoclast differentiation protocols from human induced pluripotent stem cells of different tissue origins
Source: Stem Cell Res Ther. 2023 Nov 7;14:319. doi: 10.1186/s13287-023-03547-6 (PMC10631132; doi:10.1186/s13287-023-03547-6)
Supplement: Supplementary file 1 — Additional file 1. Table S1. Antibody list. [file 13287_2023_3547_MOESM1_ESM.docx]

| Antibodies | Catalog number | Company | Dilution |
| --- | --- | --- | --- |
| Flow cytometry marker antibodies |  |  |  |
| Anti-CD34 PE-Cy7-conjugated | 560710 | BD | 1:25 |
| Anti-CD43 PerCP-Cy5.5-conjugated | 563521 | BD | 1:25 |
| Anti-CD45 APC-conjugated | 555485 | BD | 1:6 |
| Anti-CD14 BV711-conjugated | 563372 | BD | 1:25 |
| Anti-CD11b PE-Cy5-conjugated | 301308 | Biolegend | 1:25 |
| Anti-CD265/RANK PE-conjugated | FAB683P | R&D Systems | 1:12 |
| Flow cytometry isotype antibodies |  |  |  |
| Mouse IgG1, κ PE-Cy7-conjugated | 557872 | BD | 1:25 |
| Mouse IgG1, κ PerCP-Cy5.5-conjugated | 550795 | BD | 1:25 |
| Mouse IgG1, κ APC-conjugated | 555751 | BD | 1:6 |
| Mouse IgG2b, κ BV711-conjugated | 563125 | BD | 1:25 |
| Mouse IgG1, κ PE-Cy5-conjugated | 400118 | Biolegend | 1:25 |
| Mouse IgG_1_ PE-conjugated | IC002P | R&D Systems | 1:12 |
| Immunostaining |  |  |  |
| Anti-Human Otx2 NL557-Conjugated Goat IgG | SC022 | R&D Systems | 1:10 |
| Anti-Human SOX1 NL493-Conjugated Goat IgG | SC022 | R&D Systems | 1:10 |
| Anti-Human Brachyury NL557-Conjugated Goat IgG | SC022 | R&D Systems | 1:10 |
| Anti-Human HAND1 NL637-Conjugated Goat IgG | SC022 | R&D Systems | 1:10 |
| Anti-Human GATA-4 NL493-Conjugated Goat IgG | SC022 | R&D Systems | 1:10 |
| Anti-Human SOX17 NL637-Conjugated Goat IgG | SC022 | R&D Systems | 1:10 |
| Anti-Cathepsin K | ab19027 | Abcam | 1:100 |
| Goat anti-rabbit IgG H&L Alexa Fluor 647-conjugated | ab150079 | Abcam | 1:200 |

**Table 1.** Antibody list
