# Supplementary material for: Comparison of osteoclast differentiation protocols from human induced pluripotent stem cells of different tissue origins
Source: Stem Cell Res Ther. 2023 Nov 7;14:319. doi: 10.1186/s13287-023-03547-6 (PMC10631132; doi:10.1186/s13287-023-03547-6)
Supplement: Supplementary file 2 — Additional file 2. Table S2. Primer list. [file 13287_2023_3547_MOESM2_ESM.docx]

| Primers | Company | Assay ID |
| --- | --- | --- |
| POU5F1 | Thermo Fisher – TaqMan Assays | Hs04260367_gH |
| CSF1R | Thermo Fisher – TaqMan Assays | Hs00911250_m1 |
| TNFRSF11A | Thermo Fisher – TaqMan Assays | Hs00921372_m1 |
| NFATC1 | Thermo Fisher – TaqMan Assays | Hs00542675_m1 |
| CA2 | Thermo Fisher – TaqMan Assays | Hs01070108_m1 |
| MMP9 | Thermo Fisher – TaqMan Assays | Hs00957562_m1 |

**Table 2.** Primer list
