# Supplementary material for: Comparison of osteoclast differentiation protocols from human induced pluripotent stem cells of different tissue origins
Source: Stem Cell Res Ther. 2023 Nov 7;14:319. doi: 10.1186/s13287-023-03547-6 (PMC10631132; doi:10.1186/s13287-023-03547-6)
Supplement: Supplementary file 3 — Additional file 3. Supplemental Fig. S1. Cell number and viability of hematopoietic cells produced by cell forming complexes in EB and MB differentiation. MB differentiation showed a significantly lower cell yield of hematopoietic cells compared to EB differentiation following normalization to the same surface area for both PBMC- and fibroblast-derived cell lines (A). Fibroblast-derived iPSCs yielded a higher number of hematopoietic cells than PBMC-derived iPSCs using either differentiation. Cell viability in EB differentiated cells was higher than in MB differentiated for both cell lines (B). (n = 3 technical replicates). [file 13287_2023_3547_MOESM3_ESM.docx]

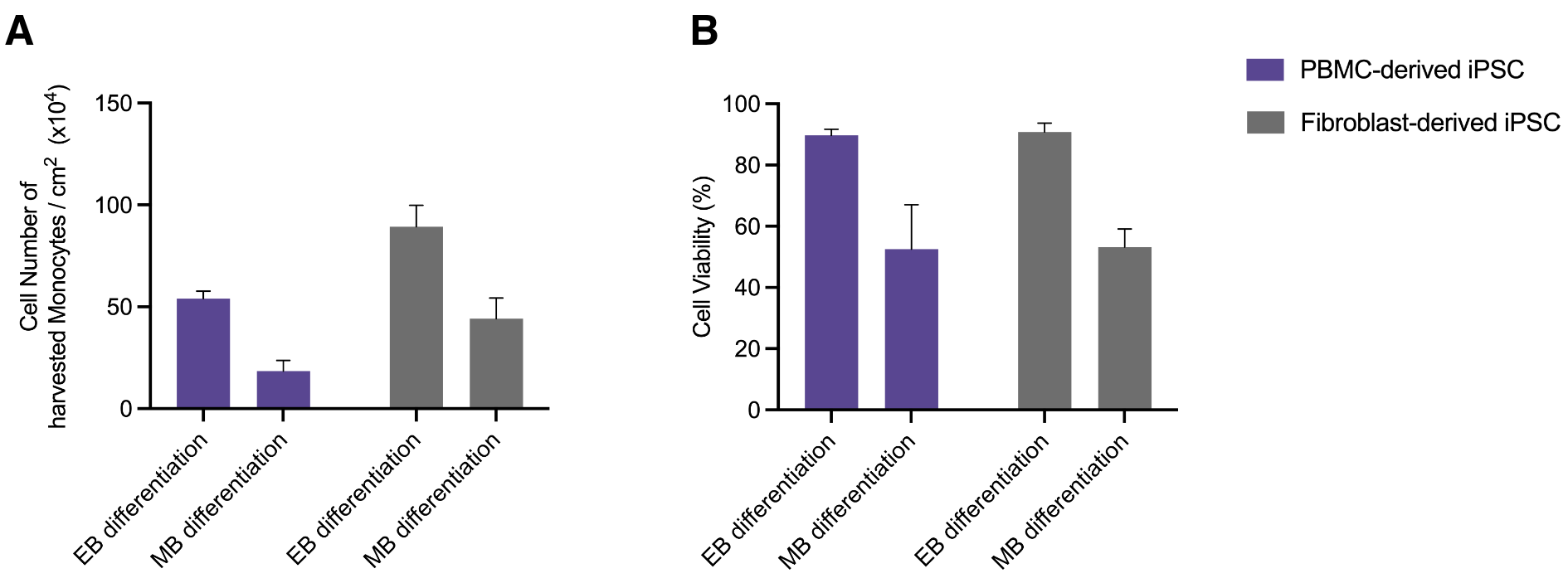


**Supplemental Fig. 1** Cell number and viability of hematopoietic cells produced by cell forming complexes in EB and MB differentiation. MB differentiation showed a significantly lower cell yield of hematopoietic cells compared to EB differentiation following normalization to the same surface area for both PBMC- and fibroblast-derived cell lines (A). Fibroblast-derived iPSCs yielded a higher number of hematopoietic cells than PBMC-derived iPSCs using either differentiation. Cell viability in EB differentiated cells was higher than in MB differentiated for both cell lines (B). (*n* = 3 technical replicates).
